# Shrinking fish: comparisons of prehistoric and contemporary salmonids indicate decreasing size at age across millennia

**DOI:** 10.1098/rsos.140026

**Published:** 2014-10-22

**Authors:** Pablo Turrero, Eva García-Vázquez, Carlos Garcia de Leaniz

**Affiliations:** 1 Department of Geology, University of Oviedo, C/ Jess Arias de Velasco, s/n. 33005, Oviedo, Asturias, Spain; 2 Department of Functional Biology, University of Oviedo, C/ Julián Clavería, s/n. 33006, Oviedo, Asturias, Spain; 3 Department of BioSciences, Swansea University, Swansea SA2 8PP, UK

**Keywords:** Atlantic salmon, back-calculation, brown trout, Palaeolithic, size at age

## Abstract

A comparison of Upper Palaeolithic and contemporary salmonid vertebrae from the Iberian Peninsula indicates that there has been a significant decrease in the mean body size for a given age among Atlantic salmon and brown trout inhabiting the southernmost range of their endemic distribution. Mean size at age was greater in prehistoric specimens for all age classes during the freshwater phase of their life histories. Fisheries-induced evolution (selection for smaller sizes) is an obvious explanation for the observed reduction in fish body size, but recent changes in the aquatic habitat affecting density-dependent growth cannot be ruled out.

## 2. Introduction

The impact of fisheries on fish abundance has been enormous owing to increasing fishing pressure and the development of more efficient fishing methods. Exploitation is a main driver of evolutionary change among wild fish populations, because fishing is seldom random with respect to heritable life-history traits [1]. For example, many fisheries are size-selective and tend to target larger than average individuals [2], whereas others may intercept individuals only during particular times, or at particular places [3]. In addition, exploited fish may have particular personality traits that can make them more or less vulnerable to fishing [4]. Inadvertent selection for smaller and younger individuals has been demonstrated for many fish species, both in marine [5,6] and freshwater [7,8] ecosystems. As a result, reproduction in many exploited stocks tends to rely on increasingly smaller and younger fish.

Methods for detecting such fisheries-induced evolution (FIE) have been developed only recently (reviewed in Kuparinen *et al*. [9]), but fish and shellfish have been a source of protein for humans for millennia. The impacts of harvesting on shellfish can be detected through significant reductions in shell size since the Upper Palaeolithic [10,11]. By contrast, size-selective exploitation of prehistoric fish has rarely been demonstrated [12]. Archaeological bone remains, however, suggest the existence of sustained high fishing pressure in some areas [13,14] and indicate that harvesting may have also been size-selective, targeting larger and older fish [15]. Analyses of prehistoric and contemporary samples are very rare, but they can provide unique insights into humans as agents of selection [16].

Here, we analyse the evolution of salmonid size in the northern Iberian Peninsula, a glacial refugium particularly rich in prehistoric fish remains and where salmonids have been exploited for over 40 000 years [17]. We compared back-calculated sizes of the only salmonids native to the region, Atlantic salmon (*Salmo salar*) and brown trout (*Salmo trutta*), fished during the Upper Palaeolithic, to contemporary samples, and estimated changes in size at age in order to infer what impact, if any, humans may have had on wild *Salmo* populations. Atlantic salmon and brown trout are facultative anadromous, and because migratory individuals tend to grow larger and faster than resident individuals [18] we consider these two life histories separately. Our expectation was that if prehistoric exploitation had been size-selective, it would have affected larger fish disproportionately more than smaller ones. We therefore expected to find a stronger shift in size at age among anadromous salmonids than among salmonids in their freshwater phase.

## 3. Material and methods

### 3.1 Archaeological material

Remains from 10 archaeological sites from the northwestern Spanish province of Asturias were searched for fish bones in the regional archaeological museum; salmonid vertebrae were then selected on the basis of their characteristic ‘honeycombed’ centra [19]. Archaeological strata age and original location of fish bones were obtained from excavation reports (more details can be found in Turrero *et al*. [20,21]).

Fish age was estimated from these vertebrae following Turrero *et al*. [20,21]. Salmonid ages are given as X.Y, where the first number refers to the number of winters spent in freshwater, and the second number, if any, refers to the number of winters spent at sea (i.e. the second number is applicable only to anadromous individuals). Salmonid species determination was not possible by visual observation of fish vertebrae alone, and both brown trout and Atlantic salmon are combined in this study, referred to as *Salmo* or *Salmo* sp.

### 3.2 Back-calculation of fish lengths

Fish length was back-calculated from prehistoric vertebrae with the help of newly developed equations relating vertebrae measurements and fork length (i.e. the distance between the snout and the fork of the tail). Back-calculation equations available in the literature usually come from predation studies [22,23] and thus tend to underestimate the size of adult specimens (because they are based on fish small enough to be eaten by predators such as otters or cormorants). The size ranges and different stocks (i.e. populations) of the specimens on which the new equations were based can be found in table 1. All specimens were measured and then dissected to obtain their vertebrae. Vertebrae measurements of fish of known fork lengths were then used to build several back-calculation equations through ordinary least-squares linear regression, which is the method recommended for archaeology and palaeoenvironmental studies [24].

**Table 1.** Size ranges in the sample used for the development of back-calculation equations. *n*, sample size; size measured is fork length.

river	species	*n*	size range (cm)	life stage
Almond	*S. salar*	14	6.5–14	juveniles
Sella	*S. salar*	78	4.5–82	juveniles, adults
Sella	*S. trutta*	58	7.5–34	juveniles, adults
Wye	*S. trutta*	2	18.5–19.5	adults

### 3.3 Contemporary samples

Data on body size and age from contemporary salmonid populations were obtained from randomly selected individuals caught by anglers in the rod and line sport fisheries (all specimens are longer than 18 cm, and all salmon are anadromous fish returning to rivers to spawn, as per current fishing legislation in the region) or caught by electrofishing during surveys (many of these carried out in the region of origin of the archaeological remains, Asturias) between 2003 and 2011.

A subset of contemporary samples were generated with 50 random vertebrae samples for each of the two salmonid species and for each of the age classes found in prehistoric samples: 1, 2, 3, 1.1, 1.2 and 1.3 years (see Results), in order to compare size at age between species. From this, a random sample (*n*=100) was taken proportionally to the age structure of the prehistoric population data in order to have comparable datasets. Because modern populations exhibit more abundant 1.1 classes [20,21], totally randomized modern samples might not contain sufficient 1.2 and 1.3 individuals for comparison purposes.

### 3.4 Data analysis

Our sample of prehistoric fish is inevitably small (*n*=32, see Results) and this precluded the fitting of von Bertalanffy growth curves to fish caught in different periods; we chose instead to compare mean size at age of prehistoric and extant fish. Length and age data were compared between pairs of samples (between Atlantic salmon and brown trout of the same age, between prehistoric and modern *Salmo*, and between salmonids from consecutive Palaeolithic periods) by means of Student's *t*-tests after checking for normality and homogeneity of variances using the PAST software v. 2.17 [25].

## 4. Results

Several back-calculation equations developed for different kinds of vertebrae are presented in table 2. Salmon and trout can sometimes be distinguished from differences in the atlases [26], but species identification is generally not possible based on other salmonid vertebrae. For this reason, equations for other types of vertebrae were developed generically for *Salmo* sp., pooling all specimens together. We estimated fork lengths using the equation from table 2 that returned the lowest mean error.

**Table 2.** Back-calculation equations developed for different types of salmonid vertebrae. Mean errors are given as percentages of actual fork lengths. FL, fork length.

measurement	equation	*n*	*R*^2^	mean error
atlas width (AW), general	FL (cm)=6.9467×AW (mm)−2.1751	152	0.9939	5.13
AW, Atlantic salmon	FL (cm)=7.0035×AW (mm)−2.1025	90	0.9968	5.07
AW, brown trout	FL (cm)=5.762×AW (mm)−0.0182	55	0.9844	6.38
‘post-atlas’ vertebra width (VW)	FL (cm)=6.6348×VW (mm)+2.9363	168	0.9577	10.01
‘post-atlas’ vertebra length (VL)	FL (cm)=8.0102×VL (mm)+2.6766	168	0.9073	14.26
thoracic VW	FL (cm)=7.0193×VW (mm)+1.364	89	0.9831	8.64
thoracic VL	FL (cm)=8.808×VL (mm)+1.6942	89	0.949	11.54
abdominal VW	FL (cm)=6.1781×VW (mm)+3.5194	65	0.9897	6.06
abdominal VL	FL (cm)=7.527×VL (mm)+0.8679	65	0.9832	6.86

A total of 32 archaeological *Salmo* vertebrae were sufficiently well preserved for age determination and back-calculation of body size. Age determination was not possible for eight vertebrae owing to taphonomic processes, but their size could be back-calculated (electronic supplementary material, table S1).

Our contemporary sample did not reveal a significant size difference between juvenile Atlantic salmon and brown trout for a given freshwater age (electronic supplementary material, table S2), brown trout measurements exhibiting high standard deviation (electronic supplementary material, table S2). The sizes of migratory age classes for both species were significantly different, migratory Atlantic salmon being significantly bigger than migratory brown trout of the same age class (as expected from the biology of the two species [18]). The *t*-tests yielded values of 12.028, 14.077 and 12.259 (*p*<0.0001 in all cases) for comparisons between 1.1, 1.2 and 1.3 Atlantic salmon and brown trout, respectively.

The prehistoric samples of North Iberian *Salmo* showed a significant decline in body size (*n*=40) and total age (*n*=32, because not all the vertebrae were useful for age determination) over time (table 3), with a drastic shift after the Magdalenian (approx. 16 000 to approx. 12 000 years BP). Fish caught in the Solutrean (approx. 20 000 to approx. 16 000 years BP) and Magdalenian periods were not significantly older (total age) than those caught in the later Epipalaeolithic (*t*=1.86, d.f.=30, *p*=0.07), but were significantly larger (*t*=3.84, d.f.=30, *p*<0.001), suggesting that there was a decrease in mean size at age for fish during the Epipalaeolithic (approx. 12 000 to approx. 6000 years BP).

**Table 3.** Mean size (±s.d.) and age (±s.d.) of *Salmo* catches from different periods in North Iberian rivers. ka, thousands of years before present; *n*: sample size. Age determinations followed Turrero *et al*. [10,21]; size was determined using the applicable equation from table 2 with the lowest mean error.

period (ka)	size (mm)	age (years)
20–16	491.24±164.12 (*n*=15)	2.4±0.74 (*n*=15)
16–12	587.20±260.99 (*n*=14)	2.38±1.06 (*n*=8)
12–9	329.24±107.22 (*n*=7)	1.8±0.84 (*n*=5)
9–6	383.41±78.79 (*n*=4)	1.75±0.96 (*n*=4)
modern	330.95±160.61 (*n*=100)	2.23±0.87 (*n*=100)

**Figure 1.**
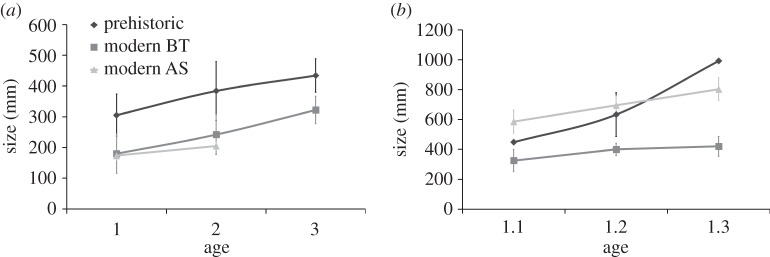
Size (mean and s.d.) of contemporary and prehistoric *Salmo* species (BT, brown trout, *S. trutta*; AS, Atlantic salmon, *S. salar*). (*a*) Freshwater phase and (*b*) marine phase.

The mean length attained during the freshwater phase was significantly smaller among contemporary Atlantic salmon and brown trout samples than among archaeological samples (table 4). Prehistoric fish were always bigger than contemporary Atlantic salmon and brown trout (figure 1*a*), with highly significant differences for 1-, 2- and 3-year-old fish despite the limited archaeological sample size (*t*-tests>3.5, *p*<0.002 in all cases). The plots of size at age for prehistoric and contemporary brown trout were parallel (contemporary Atlantic salmon do not spend more than 2 years in freshwater in the study region), suggesting that growth was similar after the first year. Young of the year (0+) individuals were not found in the archaeological samples examined.

**Table 4.** Size at age of North Iberian *Salmo* sp. specimens from different periods. ka, thousands of years before present; *n*, sample size. Modern specimens were sampled from angling catches and salmonid population surveys. Fish age is presented as X or X.Y, where X and Y are river and sea years respectively. Prehistoric lengths were back-calculated from vertebrae found in archaeological sites with the applicable equation from table 2 with the smallest mean error, and are presented as mean fork length (±s.d.).

	20–16 ka		16–12 ka		12–6 ka		modern	
fish age	*n*	length (mm)	*n*	length (mm)	*n*	length (mm)	*n*	length (mm)
1	2	368.5±56.4	2	271.7±69.8	4	290.4±59.9	25	198.4±29.2
2	5	381.5±71.9	2	457.5±121.0	1	249.6	25	240.2±63.2
1.1	0	—	0	—	2	447.9±15.6	6	405.0±142.3
3	2	449.4±18.8	1	474.1	2	447.2±8.2	16	328.7±64.7
1.2	6	628.7±158.8	2	498.1±170.2	0	—	25	510.8±151.5
1.3	0	—	1	991.9	0	—	3	556.7±203.1
total	15		8		9		100	

For prehistoric migratory *Salmo* (figure 1*b*), the average sizes of 1.1 and 1.2 individuals were intermediate between the values of the two species in the modern sample. The 1.1 individuals were significantly smaller than contemporary Atlantic salmon (*t*=11.343, d.f.=2, *p*=0.024 for samples with unequal variance) and bigger than contemporary brown trout (*t*=6.182, d.f.=4, *p*=0.003). The 1.2 prehistoric salmonids were not significantly smaller than contemporary Atlantic salmon (*t*=0.872, d.f.=16, *p*=0.405 for samples with unequal variance), but were significantly larger than contemporary brown trout (*t*=4.483, d.f.=21, *p*=0.002 for samples with unequal variance). The only 1.3 prehistoric salmonid found in the analysed archaeological sample, 99 cm back-calculated length, was bigger than any contemporary salmon or trout considered in this study.

Because there are differences in size at age between migratory Atlantic salmon and brown trout, Atlantic salmon being bigger, the size of migratory prehistoric *Salmo* does not provide information about the marine growth of Upper Palaeolithic fish, because species could not be identified from prehistoric bone remains. Any size average could be explained by different species compositions assuming equal size at age of prehistoric and contemporary migratory *Salmo* (i.e. 1.1 prehistoric individuals, of intermediate size average, could be an even mixture of Atlantic salmon and brown trout, whereas the 1.2 age class remains could contain a greater proportion of salmon than trout, thus not differing from the size average of modern 1.2 *S. salar*).

## 5. Discussion

Our comparisons of archaeological fish bones with modern samples indicate that there has been a significant reduction in the size at age of North Iberian *Salmo* since the Upper Palaeolithic, during the freshwater stage. There may be several reasons for this, including FIE resulting in smaller and younger fish, and environmental changes resulting in poorer growth. Although distinguishing between environmental constraints and the impacts of fishing is not easy [27], the fact that we have found a decrease in fish size at age suggests that fishing may have contributed, at least partially, to the observed phenotypic shift.

Strong fisheries-driven selection for smaller fish since the last glacial age [3,7,8] may explain the observed decrease in size at age of salmonids in the northern Iberian Peninsula. However, loss of habitat quality owing to siltation and pollution [28], as well as habitat fragmentation, may have also resulted in poorer contemporary growth, as barriers to migration increase local densities [29] and growth is negatively density-dependent in salmonids [30]. On the other hand, the density-independent effects of environmental factors cannot be excluded as an explanation for reduced size. Examples of these would be the temperature and dissolved oxygen content of the waters: non-preferred values of these variables would interfere negatively with salmonid growth, especially during early development [31,32]. Both hatching size and size at yolk sac reabsorption would be affected, which would be consistent with smaller sizes at age 1.

In this study, we back-calculated fish lengths from vertebrae measurements using equations specifically developed taking into account adult *Salmo*. Results obtained with the application of alternative equations [22,23,26,33,34] tend to give smaller length estimates, especially for large individuals (electronic supplementary material, table S3), most likely because those equations were developed for juvenile fish and smolts in freshwater and did not consider anadromous individuals. However, the same conclusions hold, regardless of the back-calculation equations used: mean sizes for Solutrean and Magdalenian fish calculated with juvenile equations are 467 and 408 mm, respectively, which are in any event significantly larger than the average for the modern sample; the Epipalaeolithic sample is still not significantly different from the modern one when using the juvenile equations.

Here, we have treated Atlantic salmon and brown trout together, because species identification in archaeological fish bones is inaccurate for this genus (perhaps distinguishable by the atlas [26] but only two of the remains examined here were atlases). However, we found no differences in size at age for the two species in the region during the freshwater phase (figure 1*a* and electronic supplementary material, table S1), and therefore we considered the prehistoric and modern samples to be comparable even if prehistoric ones contain a mixture of the two species. On the other hand, migratory salmon are bigger than migratory trout at the same age, and possible differences in species composition may bias comparisons between prehistoric and contemporary migratory samples (i.e. knowing that anadromous salmon are bigger on average than anadromous trout of the same age, the same average size for the genus could result from a given composition of the catch). Therefore, that differences between prehistoric and modern migratory *Salmo* are not significant does not indicate similar growth at sea *per se*, and differences in the anadromous phase of development of *Salmo*species between the Upper Palaeolithic and the present moment cannot be ruled out.

Furthermore, differences in life-history traits between Upper Palaeolithic and contemporary salmonids in the region have been found to occur during the marine phase, which has been shortened, whereas the duration of the freshwater phase would have remained more or less unchanged across millennia [20]. However, our findings suggest that growth may have decreased during the first freshwater year since the Upper Palaeolithic, and 2- and 3-year-old individuals are now smaller than in the Palaeolithic. Given that marine survival is positively related to smolt size in anadromous salmonids [35,36], reduced freshwater growth would have resulted in smaller smolts and poorer survival at sea, which may explain, at least partially, current salmon declines in this region despite increasingly restrictive fishing legislations and extensive stocking efforts [37]. Our comparisons between contemporary and Upper Palaeolithic samples suggest that historical changes in freshwater environments may have had critical carryover effects on the survival of anadromous salmonids at the southern edge of their distribution.

## Data accessibility

The electronic supplementary material comprises the following: table S1: list of prehistoric samples (chronology, age and size); table S2: mean sizes of contemporary *Salmo*; table S3: alternative size back-calculations.
